# The Association of Visceral Adiposity with Cardiovascular Events in Patients with Peripheral Artery Disease

**DOI:** 10.1371/journal.pone.0082350

**Published:** 2013-12-27

**Authors:** Oliver Cronin, Barbara Bradshaw, Vikram Iyer, Margaret Cunningham, Petra Buttner, Philip J. Walker, Jonathan Golledge

**Affiliations:** 1 Queensland Research Centre for Peripheral Vascular Disease, School of Medicine and Dentistry, James Cook University, Townsville, Queensland, Australia; 2 Discipline of Surgery and Centre for Clinical Research, School of Medicine, University of Queensland, Brisbane, Queensland, Australia; Department of Vascular Surgery, Royal Brisbane and Women's Hospital, Brisbane, Queensland, Australia; 3 School of Public Health, Tropical Medicine and Rehabilitation Sciences, James Cook University, Townsville, Queensland, Australia; 4 Department of Vascular and Endovascular Surgery, The Townsville Hospital, Townsville, Queensland, Australia; Washington Hospital Center, United States of America

## Abstract

**Background:**

Previous studies have suggested that patients with peripheral artery disease (PAD) suffer from a high incidence of cardiovascular events (CVE). Visceral adiposity has been implicated in promoting CVEs. This study aimed to assess the association of relative visceral adipose volume with incident cardiovascular events in patients with peripheral artery disease.

**Methods:**

This was a prospective cohort study including 260 patients with PAD who presented between 2003 and 2012. Cases were patients with diagnosed PAD including symptomatic lower limb athero-thrombosis and asymptomatic abdominal aortic aneurysm. All patients underwent computed tomography angiography (CTA). Abdominal visceral to total adipose volume ratio (relative visceral adipose volume) was estimated from CTAs using a previously validated workstation protocol. Cardiovascular risk factors were recorded at entry. The association of visceral adiposity with major CVEs (death, non-fatal myocardial infarction or stroke) was examined using Kaplan Meier and Cox proportional hazard analyses.

**Results:**

A total of 92 major CVEs were recorded in 76 patients during a median follow-up of 2.8 (IQR 1.2 to 4.8) years, including myocardial infarction (n = 26), stroke (n = 10) and death (n = 56). At 3 years the incidence of major CVEs stratified by relative visceral adipose volume quartiles were 15% [Quartile (Q) 1], 17% (Q2), 11% (Q3) and 15% (Q4) (*P* = 0.517). Relative visceral adipose volume was not associated with major CVEs after adjustment for other risk factors.

**Conclusion:**

This study suggests that visceral adiposity does not play a central role in the predisposition for major CVEs in patients with PAD.

## Introduction

Patients with peripheral artery disease (PAD) [i.e. stenosing or aneurysmal disease outside the coronary circulation] have a high incidence of cardiovascular events (CVEs) [1]–[4]. The incidence of major CVEs in patients with PAD has been reported in some studies to be higher than in patients with athero-thrombosis affecting other vascular sites, such as coronary heart disease [5],[6].

Current management of CVE risk reduction in PAD patients is limited with a number of previous studies reporting that patients with PAD are frequently managed less intensively than patients with athero-thrombosis at other sites despite a high incidence of major CVEs [7]–[9]. There is considerable interest in identifying modifiable risk factors that could decrease the high incidence of CVEs seen in patients with PAD [1]. Obesity is an established risk factor for cardiovascular disease and CVEs in patients with coronary heart disease [10], [11]. A recent systematic review suggested that obesity was associated with cardiovascular events in patients with PAD [12]. Adipose is deposited at both subcutaneous and visceral sites [13]. It has been suggested that visceral adipose may have a unique composition of white relative to brown adipose that has been implicated in the secretion of pro-inflammatory cytokines which could promote CVEs [14]. Reduction of visceral adipose could provide a new avenue for risk reduction [2]. For example previous studies have reported that visceral adipose can be targeted by exercise regimens and thus visceral adiposity is potentially a modifiable risk factor [15], [16].

Markers of visceral adiposity, such as waist circumference (WC) and waist to hip ratio (WHR) have been independently associated with the severity and prognosis of lower limb atherosclerosis [2], [17]. The measures of visceral adiposity previously used do not however directly assess the amount of visceral adipose tissue. We have previously designed and validated a method to measure visceral adipose volume from thresholded computed tomographic angiography (CTA) images [18]. The association of visceral adipose volume with incident CVEs in patients with PAD has not been previously assessed. The aim of this study was to assess the association between visceral adipose volume and incident major CVEs in patients with PAD.

## Methodology

### Study Participants and Definitions

This research was approved by the Townsville Health Service District Human Research Ethics Committee. This was a prospective cohort study of patients who presented to The Townsville Hospital vascular clinic between May 2003 and February 2012. Patients presented to clinic for investigation or surveillance of PAD. Patients were assessed by a vascular surgeon that diagnosed PAD based on appropriate symptoms and signs of lower limb athero-thrombosis and CTA imaging evidence of lower limb atherosclerosis or abdominal aortic aneurysm (AAA). AAA was defined as an infra-renal aortic diameter ≥30 mm. All included patients were diagnosed with lower-limb athero-thrombosis, AAA or both. Inclusion criteria were as follows: 1) Verbal and written consent; and 2) a clinical need for CTA. Exclusion criteria were: 1) Urgent requirement for peripheral vascular surgical intervention; 2) contra-indication to CTA. There were no further inclusion or exclusion criteria.

### Computed tomography angiography analysis

All CTAs were performed at The Townsville Hospital using a 64-slice multi-scanner (Philips, North Ryde, NSW) under a set protocol. Images were recorded at 3 mm intervals with a slice thickness of 3 mm enabling the construction of 3 mm adjoining axial images for analysis. One hundred millimetres of Ultravist 300 contrast agent was administered intra-venously by an automatic CTA injection driver system (MEDRAD). The CTA imaging commenced once the Hounsfield Unit (HU) at the centre of the aorta reached 130. CTA imaging was transferred to Philips MxView Visualisation Workstation software for analysis. The two researchers performing measurements underwent a comprehensive training program provided by a clinician experienced in use of the workstation.

### Assessment of abdominal adipose volume

A protocol was designed which combined techniques described in published literature with approaches learnt whilst performing aortic volumetric analysis [19]–[23]. This protocol has been previously described [18]. Briefly, the radiographic threshold was set to the pre-defined threshold for adiposity (CH −120 HU, WH 75 HU). The axial slice at which the superior aspect of the first sacral body appeared was identified. The “Volume of Interest” tool was used to manually circumscribe the entire torso at this slice. The axial slice 125 mm cephalad to the first slice was identified and circumscribed with the “Volume of Interest” tool. In a semi-automated system, the axial slices between these points were methodically joined to form a deposit of adipose tissue. “Tissue Volume” was selected to provide the total adipose tissue volume.

In a similar process the visceral adipose volume was calculated from axial CTA images. Firstly the visceral compartment at the most superior aspect of the first sacral body was manually traced using the “Volume of Interest” tool. Secondly the visceral compartment on the axial slice 125 mm cephalad was circumscribed. The axial slices between these points were joined in a semi-automated fashion to create an estimation of the visceral adipose volume. Subcutaneous adipose volume was calculated from the difference between total and visceral adipose volumes. Measurements were displayed to the nearest 0.01 cm^3^ [18].

### Reproducibility analysis for adipose volume

A reproducibility analysis of the first 15 participants recruited into the study was completed by two observers as previously reported [18]. Intra-observer reproducibility measurements were completed three times with at least one week in between measurement sets. Inter-observer reproducibility was completed at least one week after the intra-observer measurements were complete. Data was analysed by a third independent observer. The intra- and inter-observer concordance correlation coefficients (CCC) for total adipose volume measurements were 1.0 (95% CI, 1.0 to 1.0) and 1.0 (95% CI, 1.0 to 1.0) respectively. The intra- and inter-observer average coefficients of variation (ACV) for total adipose volume were 0.8% and 1.2% respectively. The CCCs for intra- and inter-observer visceral adipose volumes were 1.0 (1.0 to 1.0) and 1.0 (1.0 to 1.0) respectively. The ACV for intra- and inter-observer visceral adipose volume were 1.9% and 3.4% respectively.

### Clinical data

Baseline characteristics collected for each patient at entry to the study included: Gender; age; hypertension; diabetes; coronary heart disease and medication prescription. Diabetes and hypertension were defined by history of diagnosis or treatment of these conditions. History of smoking was defined as ever or never smoked. Coronary heart disease was defined by a history of angina, myocardial infarction or coronary revascularisation. Current medication history was recorded with respect to prescription of angiotensin converting enzyme (ACE) inhibitors, angiotensin receptor blockers, aspirin, beta-blockers, calcium channel blockers, other anti-platelet medication, statins and warfarin.

### Follow-up for cardiovascular events

After the initial assessment and imaging the frequency of patient review was dependent on the patient's presenting complaint. Patients with intermittent claudication were typically reviewed six months after entry to the study and annually thereafter. Patients with small AAAs (i.e. 30–39 mm) were typically reviewed annually and patients with larger AAAs (i.e. ≥40 mm) were typically reviewed bi-annually.

The primary outcome measure in this study was the incidence of major CVEs including non-fatal myocardial infarction, non-fatal stroke or death. Secondary outcome measures were non-fatal myocardial infarction and non-fatal stroke. Outcome data was recorded either at clinic appointments or during hospital admissions. Cause of death was verified by the death certificate where possible. Patient charts were reviewed to ensure hospital admissions were not missed.

### Statistical analysis

Based on previous studies we estimated over an average follow-up of approximately three years that the incidence of death, myocardial infarction or stroke would be 30% [2], [24]. We planned to perform a Cox proportional analysis to assess the association of visceral adiposity with cardiovascular events adjusted for the following risk factors: Age, gender, coronary heart disease, diabetes, hypertension and smoking. Thus we estimated that we needed to include approximately 230 patients in order to achieve 10 outcome events per risk factor included in the planned Cox regression model. Previous studies suggest this approach provides sufficient power to assess this number of dependent variables [25]. Data were prospectively recorded in an Access database and subsequently transferred to a Microsoft Excel spread sheet and then to SPSS Version 20.0 (IBM SPSS Inc., Chicago, Illinois) for statistical analysis. Abdominal visceral to total adipose volume ratio (relative visceral adipose volume) was calculated for all cases. Participants were stratified into quartiles [Quartiles (Q) 1 to 4] determined by their relative visceral adipose volume in ascending order for analysis. Histograms and Kolmogorov-Smirnov tests demonstrated that numerical data were not normally distributed. Statistical methods of survival analysis were applied to assess occurrence of CVE during follow-up. Follow-up time was defined as the time between the date of the initial CTA and occurrence of the first CVE or last observation. Kaplan Meier analysis was used to estimate cumulative survival probability for CVE incidence. The independent association of visceral adiposity with the incidence of CVEs was assessed by Cox proportional hazard analysis adjusting for established risk factors, including age, coronary heart disease, diabetes, gender, hypertension and smoking history. Results of the Cox analysis are presented as hazard ratios (HR) and 95% confidence intervals (95%-CI). Only the first event was used in the Cox analysis if more than one event was recorded for the participant.

## Results

### Characteristics of patients at recruitment

From May 2003 to February 2012 260 participants were recruited. All study participants resided in north Queensland, mostly from Townsville and surrounding communities. Table 1 illustrated the presenting characteristics of the patients in relation to the quartile of relative visceral adipose volume defined as Q1 (lowest relative visceral adipose volume) to Q4 (highest relative visceral adipose volume). The study group comprised 106 (41%) cases with AAA, 128 (49%) cases with lower limb athero-thrombosis and 26 (10%) cases with both AAA and athero-thrombosis. Median relative visceral adipose volumes in Q1, Q2, Q3 and Q4 were 0.26 [inter-quartile range (IQR) 0.20 to 0.32], 0.43 (IQR 0.41 to 0.45), 0.52 (IQR 0.49 to 0.56) and 0.64 (IQR 0.61 to 0.70) respectively. At entry higher relative visceral adipose volume was noted in patients who were male, had diabetes, had a larger WC and had larger abdominal aortas (Table 1). All other characteristics were similar between quartiles of relative visceral adipose volume.

**Table 1:** Association of entry cardiovascular risk factors with visceral adiposity at the time of CTA imaging.

Relative visceral adipose volume					
Characteristic	Quartile 1 (n = 65)	Quartile 2 (n = 65)	Quartile 3 (n = 65)	Quartile 4 (n = 65)	*P* value
Age (years)	70 (62–76)	68 (61–75)	72 (65–76)	70 (64–76)	0.188
Body Mass Index (kg/m^2^)	26.8 (23.7–29.9)	28.7 (24.2–31.1)	28.7 (25.1–31.4)	28.4 (26.1–31.7)	0.240
Coronary Heart Disease	28 (43)	38 (58)	31 (48)	38 (58)	0.193
*Diabetes mellitus*	*15 (23)*	*18 (28)*	*21 (32)*	*30 (46)*	*0.031*
Ever smoked	55 (85)	54 (83)	58 (89)	59 (91)	0.512
History of Stroke	6 (9)	6 (9)	4 (6)	11 (17)	0.219
Hypertension	50 (77)	46 (71)	50 (77)	57 (88)	0.131
*Male gender*	*16 (25)*	*53 (82)*	*58 (89)*	*65 (100)*	*<0.001*
*Waist Circumference (cm)*	*95.5 (80.5–105.8)*	*98.0 (84.0–108.0)*	*105.0 (92.0–118.0)*	*106.0 (92.0–118.0)*	*0.016*
Abdominal aortic aneurysm	19 (29)	25 (38)	33 (51)	29 (45)	0.078
Intermittent claudication	40 (62)	32 (49)	27 (42)	29 (45)	0.110
AAA & IC	6 (9)	8 (12)	5 (8)	7 (11)	0.836
Medications					
Aspirin	43 (66)	46 (71)	42 (65)	45 (69)	0.872
ACE inhibitor	21 (32)	31 (48)	26 (40)	29 (45)	0.307
Angiotensin receptor blocker	17 (26)	8 (12)	9 (14)	16 (25)	0.092
Beta-Blocker	25 (38)	29 (45)	18 (28)	24 (37)	0.251
Calcium channel blocker	22 (34)	16 (25)	22 (34)	24 (37)	0.469
Other anti-platelet	9 (14)	11 (17)	8 (12)	12 (18)	0.757
Statin	40 (62)	44 (68)	36 (55)	41 (63)	0.544
Warfarin	7 (11)	8 (12)	5 (8)	5 (8)	0.754
Cardiovascular Outcomes					
Follow-up (years)	2.7 (1.0–4.6)	3.7 (1.3–5.5)	2.3 (1.0–4.0)	2.9 (1.6–4.8)	0.306
Myocardial infarction	9 (14)	8 (12)	1 (2)	8 (12)	0.072
Stroke	3 (5)	2 (3)	3 (5)	2 (3)	0.937
Death	16 (25)	11 (17)	14 (22)	15 (23)	0.735
≥1 event*	22 (34)	17 (26)	16 (25)	21 (32)	0.586
**Computed Tomography Imaging Morphology**					
**Infra-renal aorta measurements**					
*Maximum orthogonal φ (mm)*	*23.3 (19.3–41.4)*	*29.8 (21.7–45.2)*	*43.5 (23.2–55.9)*	*35.7 (24.3–48.1)*	*<0.001*
*Total volume (cm^3^)*	*35.1 (22.1–90.6)*	*46.0 (27.9–107.0)*	*98.3 (32.0–153.1)*	*77.0 (35.5–115.6)*	*<0.001*
**Abdominal Fat Measurements**					
*Total volume (cm^3^)*	*3895.8 (2145.8–5835.9)*	*3655.1 (2247.6–4938.4)*	*4047.5 (3238.2–5184.7)*	*4865.9 (3803.0–5641.0)*	
*Visceral volume (cm^3^)*	*909.1 (380.0–1558.0)*	*1654.3 (927.7–2058.9)*	*2169.6 (1675.0–2809.1)*	*3112.3 (2562.2–3773.4)*	,
*Relative visceral adipose volume*	*0.258 (0.203–0.319)*	*0.432 (0.407–0.450)*	*0.524 (0.494–0.555)*	*0.639 (0.607–0.704)*	

Categorical variables are presented as numbers (%) and compared by Chi-square tests. Numerical variables are presented as median (inter-quartile range) and compared by Kruskal-Wallis tests. AAA = Abdominal aortic aneurysm; IC = intermittent claudication; ACE = Angiotensin converting enzyme; φ = Diameter.

≥1 event (stroke, myocardial infarction or death). Relative visceral adipose volume = visceral-to-total abdominal adipose volume ratio. Quartiles are stratified by relative visceral adipose volume in ascending order. Body mass index and waist circumference data was missing for 10 and 99 patients respectively. The significance level is 0.05. *Italicised* font denotes significance.

### Kaplan Meier Analysis

**Figure 1:**
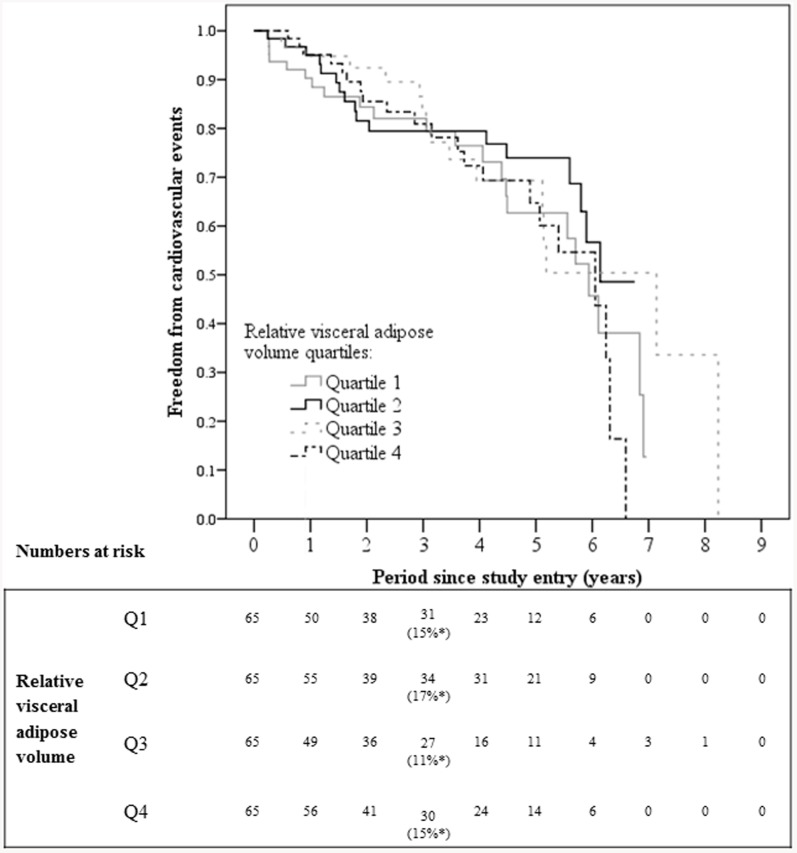
Kaplan Meier analysis illustrating freedom from cardiovascular events in relation to relative visceral adipose volume quartiles. There was no significant association between the incidence of non-fatal myocardial infarction, non-fatal stroke or death and visceral adiposity. *Cardiovascular event incidence at 3 years (*P* = 0.517).

Patients were followed for a median of 2.8 years (IQR 1.2 to 4.8) from the initial CTA until the first event or loss to follow-up. A total of 92 major CVEs were recorded in 76 patients. The major CVEs included myocardial infarction (n = 26), stroke (n = 10) and death (n = 56). Overall the combined incidence of myocardial infarction, stroke or death was 15% at 3 years. Higher relative visceral adipose volume was not associated with the cumulative incidence of major CVEs (Figure 1). At 3 years the incidence of major CVEs was 15%, 17%, 11% and 15% for patients with relative visceral adipose volume in quartiles one, two, three and four, respectively, at entry, *P* = 0.517. The incidence of non-fatal myocardial infarction and non-fatal stroke were also not associated with relative visceral adipose volume quartiles (Figure S1 and Figure S2). The relationship between visceral adiposity and the combined incidence of myocardial infarction, stroke or death were also analysed separately for the following sub-sets of patients with no significant associations found: Patients with and without diabetes (Figure S3 and Figure S4); patients with AAA (Figure S5) and patients with lower limb athero-thrombosis but no AAA (Figure S6).

### Cox proportional hazard analysis

Relative visceral adipose volume was not associated with combined major CVEs after adjustment for traditional risk factors (Table 2). Older age (HR 2.98; 95%CI 1.76 to 5.04) and coronary heart disease (HR 1.76; 95%CI 1.03 to 3.00) were associated with the incidence of combined major CVEs. Separate analyses for non-fatal myocardial infarction and stroke are illustrated in Table S1 and Table S2. There was no consistent association of visceral adipose volumes with non-fatal myocardial infarction although patients in quartile three did appear to have a lower incidence of events compared to those in quartile one (HR 0.094; 95% CI 0.010 to 0.892) (Table S1). No association was found between traditional cardiovascular risk factors and non-fatal stroke incidence (Table S2). All patients who had non-fatal stroke had a smoking history and hence the data was multi-collinear. The data was re-analysed to exclude those participants without a smoking history but the results were similar (not shown). Relative visceral adipose volume was not associated with non-fatal stroke incidence (Table S2). Separate analyses of the following sub-sets of patients did not find any association between relative visceral adipose volume and combined CVEs after adjustment for traditional risk factors: Patients with and without diabetes; patients with AAA and patients with lower limb athero-thrombosis but no AAA (Table S3, Table S4, Table S5 and Table S6).

**Table 2:** Independent determinants of cardiovascular events (myocardial infarction, stroke or death) in patients with PAD.

Prognostic Factor	Sample Size (n = 260)	Cardiovascular Events (n = 76)	HR (95% C.I.)	*P* Value
Relative visceral adipose volume				
Quartile 1	65	22	1 (Ref.)	
Quartile 2	65	17	1.111 (0.520–2.371)	0.786
Quartile 3	65	16	1.035 (0.449–2.387)	0.935
Quartile 4	65	21	1.228 (0.509–2.960)	0.647
Age				
Below median	126	24	*1 (Ref.)*	
Above Median	134	52	*2.981 (1.762–5.044)*	*<0.001*
Coronary Heart Disease				
Absent	125	26	*1 (Ref.)*	
Present	135	50	*1.758 (1.032–2.995)*	*0.038*
Diabetes				
Absent	176	49	1 (Ref.)	
Present	84	27	1.025 (0.620–1.697)	0.922
Gender				
Female	68	26	1 (Ref.)	
Male	192	50	1.858 (0.901–3.829)	0.093
Hypertension				
Absent	57	14	1 (Ref.)	
Present	203	62	0.899 (0.463–1.745)	0.753
Smoking History				
Absent	34	7	1 (Ref.)	
Present	226	69	2.020 (0.876–4.656)	0.099

HR = hazard ratio, CI = confidence interval, Ref. = reference. Relative visceral adipose volume = visceral-to-total abdominal adipose volume ratio. Quartiles are stratified by relative visceral adipose volume in ascending order. The significance level is 0.05. *Italicised* font indicates significance.

## Discussion

The main finding from this study was that relative abdominal visceral adipose volume assessed from thresholded CTA was not associated with major CVEs in patients with PAD. The incidence of major CVEs in this study was similar to other comparative studies such as Alberts and colleagues who reported a 3 year incidence for major CVEs of 6% and 12% for asymptomatic and symptomatic PAD cases respectively [4].

Eight studies have previously assessed the association of various anthropometric measures with CVEs in PAD populations (Table 3) [2], [5], [6], [17], [18], [26]–[28] however no previous studies specifically assessed abdominal visceral adipose volume. Of the eight identified studies, two studies reported a more powerful association of WC with CVEs than body mass index (BMI) [2], [17]. Results from the studies that assessed BMI were conflicting: two studies reported less CVEs in obese subjects [5], [18]; one study reported a negative association of overweight but not obesity with CVEs [28], one study reported an inverse association of BMI>20 kg/m^2^ with CVEs [6] and one study did not find a significant association between BMI and CVEs [27]. One study did not find a significant association between WHR and CVEs [26]. Although only based on two studies, these results suggest that WC may be a more consistent predictor of CVEs than BMI in PAD patients [2], [17]. WC is a commonly used estimate of visceral adipose although it does not directly measure visceral adipose volume [29].

**Table 3:** Summary of studies assessing the association of obesity and cardiovascular events in patients with peripheral artery disease [12].

Study	N with PAD	Obesity Measure	Outcome Event	Median Follow-up, years	HR	95%CI	P	Conclusion
Barba et *al.* (2009) [28]	724	BMI≥20 kg/m^2^	Combined events: MI, ischaemic stroke, critical limb ischaemia, cardiovascular death	1.2	-	-	-	Inverse association between BMI and cardiovascular mortality
Bhatt et *al.* (2010) [6]	7191	BMI>30 kg/m^2^	Combined events: Cardiovascular death, MI, stroke, cardiovascular hospitalisation	4.0	-	-	-	BMI<20 had a higher incidence of major CVEs compared to BMI>20
Diehm et *al.* (2009) [27]	1 429	BMI≥30 kg/m^2^	Combined events: MI, coronary revascularization, stroke, carotid revascularization, or lower-extremity peripheral vascular events	5.0	1.05	0.90–1.22	-	BMI>30 kg/m^2^ was not associated with death or severe CVEs
Giugliano et *al.* (2010) [17]	190	BMI≥30 kg/m^2^; WC≥88 cm women, ≥102 cm male	Combined events: MI, angina, coronary revascularisation, Cerebrovascular event, peripheral limb ischaemia, cardiovascular death	2.6	1.08*	1.01–1.15	0.045	WC associated with CVEs but not BMI. Abdominal obesity and to a lesser extent general obesity confers a worse prognosis
Golledge et *al.* (2007) [2]	60	WC>80 cm female, >94 cm male; Additional measures: BMI (kg/m^2^); WHR	Combined events: Death, MI, stroke, coronary or peripheral revascularisation	2.0	1.16	1.08–1.26	<0.001	WC was associated with cardiovascular events
Golledge et *al.* (2013) [18]	1 472	BMI≥30 kg/m^2^	Death	1.4	0.59	0.41–0.85	0.005	Obesity associated with a reduced risk of death
Lakshmanan et *al.* (2010) [26]	193	WHR>0.9	Cardiovascular death	5.7	0.93	0.72–1.20	-	Cardiovascular mortality was not associated with WHR>0.9
Reid et *al.* (2012) [5]	256	BMI≥30 kg/m^2^	Combined events: Death, MI, stroke, hospitalisation for cardiac procedure	1.0	-	-	-	Obese cases had a lower incidence of major CVEs compared to overweight and ideal BMI cases

PAD, peripheral artery disease; HR, hazard ratio; CI, confidence interval; WC, waist circumference; BMI, body mass index; WHR, waist-to-hip ratio; MI, myocardial infarction; CVE, cardiovascular event;

Model incorporating BMI and waist circumference.

Two studies have previously assessed the association of visceral adipose measured from thresholded CTAs with CVEs although these were not in patients with PAD [30], [31]. Gabriella and colleagues assessed a group of HIV positive patients and Kamimura and colleagues assessed a group of patients with chronic kidney disease [30], [31]. Both studies reported an association between visceral adipose and cardiovascular death [30], [31]. BMI was not associated with CVEs [30]. However Kamimura and colleagues assessed visceral adiposity by area from axial slices rather than volume as has been reported in this study [31]. Volumetric assessment could be suggested as a more accurate estimate of visceral adiposity. Both studies used 10 mm thick axial slices for assessment [30], [31] compared to this study which used 3 mm axial slices to provide a more detailed abdominal examination.

The findings in the current study differ from the findings of previous studies which have reported an association between WC and CVEs [2], [17] however our method directly assessed visceral adipose volume rather than other surrogate anthropometric measures which are more commonly used. We postulated that visceral adipose may be an important contributor to CVEs in patients with PAD. The majority of arteries including the abdominal aorta are surrounded by peri-vascular adipose tissue [14]. Visceral adipose has been shown to release pro-inflammatory adipokines which may promote adverse CVEs [14], [32]–[34]. However our findings suggest that other modifiable risk factors may play a more powerful role in promoting CVEs in PAD patients.

Several studies have reported an ‘obesity paradox’ in cohorts of patients with chronic disease such as heart failure [5], [18], [35]–[37]. Higher BMIs (≥30.0 kg/m^2^) have been associated with lower mortality; overweight (25.0 to 29.9) kg/m^2^ and obese cases had a lower risk of death relative to healthy BMI (18.5 kg/m^2^ to 24.9) kg/m^2^ controls [5], [18], [35]–[37]. Reid and colleagues reported that at 1 year the incidence of major CVEs in PAD cases was higher in the normal weight group (11.6%) compared to the overweight (10.9%) and obese groups (9.3%) [5]. While the underlying mechanisms of the ‘obesity paradox’ remain unclear [5] it appears likely the contradictory factors like nutritional status, muscle mass and association of other risk factors such as physical activity maybe important [2], [17], [18]. The findings of the current study suggest that complications of PAD maybe better limited by lifestyle modification targeting smoking cessation and increased physical activity [38] and medical therapies (ACE inhibitors [39], beta-blockers, aspirins and statins) rather than therapies focused on adipose distribution *per se* [40].

This study has a number of limitations. Firstly considering the need to adjust for confounding variables the sample size in this study was relatively small but in line with our sample size calculation. Secondly, patients were from a mixed group with either AAA, lower limb athero-thrombosis or both. CTA imaging of a ‘healthy’ control group would not have been ethical or appropriate. Characteristics of the included participants were however similar considering they had similar risk factors. Thirdly measurements of visceral adiposity were limited to the abdomen and did not include visceral adiposity from other sites.

In conclusion this study suggests that relative visceral adipose volume is not associated with major CVEs in patients with PAD. These findings do not support interventions specifically targeting visceral adipose as secondary preventative measures in patients with PAD.

## Supporting Information

Figure S1 **Kaplan Meier analysis illustrating freedom from myocardial infarction in relation to relative visceral adipose volume quartiles.** There was no significant association between the incidence of myocardial infarction and visceral adiposity. *Myocardial infarction event incidence at 3 years (*P* = 0.072).
(TIF)

Figure S2 **Kaplan Meier analysis illustrating freedom from stroke in relation to relative visceral adipose volume quartiles.** There was no significant association between the incidence of stroke and visceral adiposity. *Stroke incidence at 3 years (*P* = 0.857).
(TIF)

Figure S3 **Kaplan Meier analysis illustrating freedom from cardiovascular events in relation to relative visceral adipose volume quartiles in patients with diabetes at entry.** There was no significant association between the incidence of non-fatal myocardial infarction, non-fatal stroke or death and visceral adiposity. *Cardiovascular event incidence at 3 years (*P* = 0.432).
(TIF)

Figure S4 **Kaplan Meier analysis illustrating freedom from cardiovascular events in relation to relative visceral adipose volume quartiles in patients that did not have diabetes at entry.** There was no significant association between the incidence of non-fatal myocardial infarction, non-fatal stroke or death and visceral adiposity. *Cardiovascular event incidence at 3 years (*P* = 0.297).
(TIF)

Figure S5 **Kaplan Meier analysis illustrating freedom from cardiovascular events in relation to relative visceral adipose volume quartiles in patients with AAA at entry.** There was no significant association between the incidence of non-fatal myocardial infarction, non-fatal stroke or death and visceral adiposity. *Cardiovascular event incidence at 3 years (*P* = 0.093).
(TIF)

Figure S6 **Kaplan Meier analysis illustrating freedom from cardiovascular events in relation to relative visceral adipose volume quartiles in patients that did not have an AAA at entry.** There was no significant association between the incidence of non-fatal myocardial infarction, non-fatal stroke or death and visceral adiposity. *Cardiovascular event incidence at 3 years (*P* = 0.480).
(TIF)

Table S1 Independent determinants of non-fatal myocardial infarction in patients with PAD.
(DOCX)

Table S2 Independent determinants of non-fatal stroke in patients with PAD.
(DOCX)

Table S3 Independent determinants of cardiovascular events (myocardial infarction, stroke, death) in patients with PAD and diabetes mellitus.
(DOCX)

Table S4 Independent determinants of cardiovascular events (myocardial infarction, stroke, death) in patients with PAD *without* diabetes mellitus.
(DOCX)

Table S5 Independent determinants of cardiovascular events (myocardial infarction, stroke, death) in patients with AAA.
(DOCX)

Table S6 Independent determinants of cardiovascular events (myocardial infarction, stroke, death) in patients with PAD *excluding AAA patients*.
(DOCX)
