# Supplementary figures and images for: The Association of Visceral Adiposity with Cardiovascular Events in Patients with Peripheral Artery Disease
Source: PLoS One. 2013 Dec 27;8(12):e82350. doi: 10.1371/journal.pone.0082350 (PMC3873921; doi:10.1371/journal.pone.0082350)

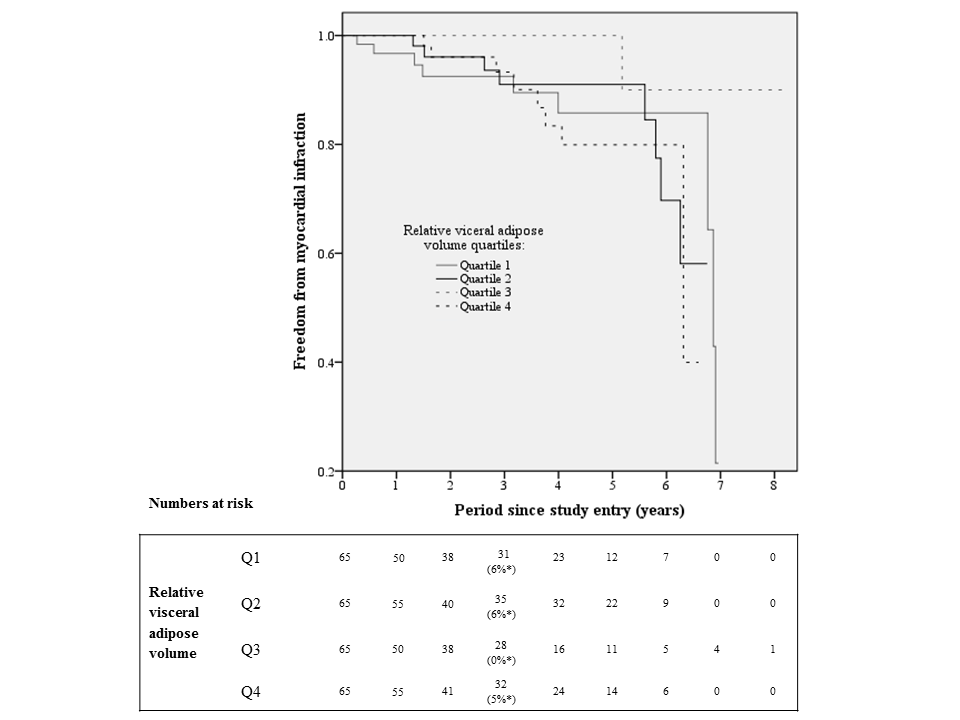

Supplement: Figure S1 — Kaplan Meier analysis illustrating freedom from myocardial infarction in relation to relative visceral adipose volume quartiles. There was no significant association between the incidence of myocardial infarction and visceral adiposity. *Myocardial infarction event incidence at 3 years (P = 0.072). (TIF) [file pone.0082350.s001.tif]

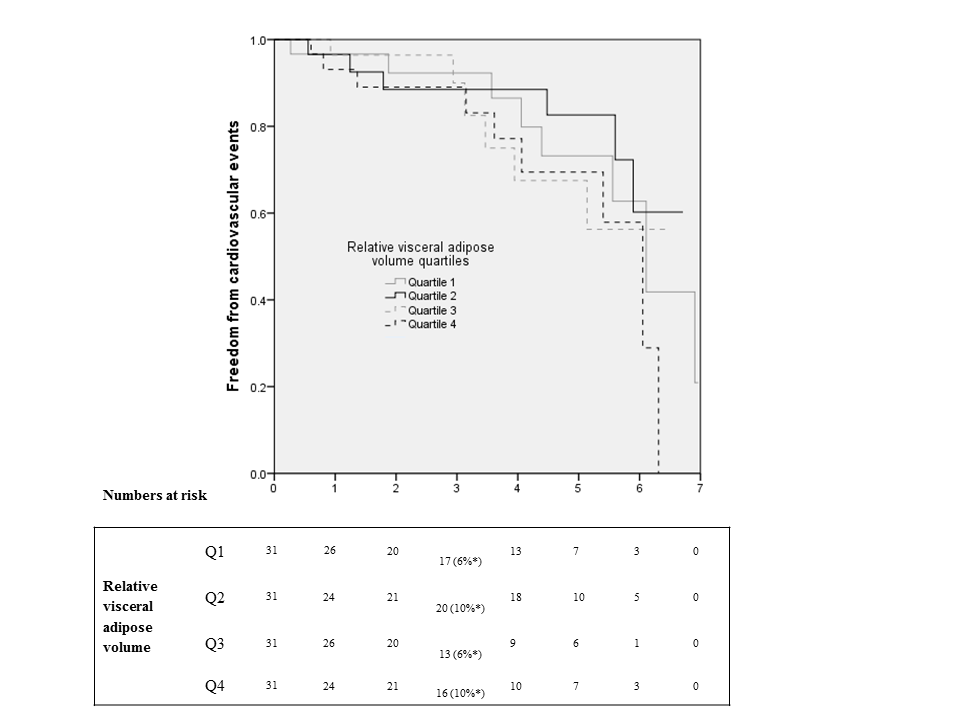

Supplement: Figure S6 — Kaplan Meier analysis illustrating freedom from cardiovascular events in relation to relative visceral adipose volume quartiles in patients that did not have an AAA at entry. There was no significant association between the incidence of non-fatal myocardial infarction, non-fatal stroke or death and visceral adiposity. *Cardiovascular event incidence at 3 years (P = 0.480). (TIF) [file pone.0082350.s006.tif]
