# Supplementary material for: The Association of Visceral Adiposity with Cardiovascular Events in Patients with Peripheral Artery Disease
Source: PLoS One. 2013 Dec 27;8(12):e82350. doi: 10.1371/journal.pone.0082350 (PMC3873921; doi:10.1371/journal.pone.0082350)
Supplement: Table S1 — Independent determinants of non-fatal myocardial infarction in patients with PAD. (DOCX) [file pone.0082350.s007.docx]

**Table S1: Independent determinants of non-fatal myocardial infarction in patients with PAD.**

| **Prognostic Factor** | **Sample Size (n=260)** | **Myocardial Infarction (n=26)** | **HR (95% C.I.)** | ***P* Value** |
| --- | --- | --- | --- | --- |
| Relative visceral adipose volume |  |  |  |  |
| Quartile 1 | 65 | 9 | 1 (Ref.) |  |
| Quartile 2 | 65 | 8 | 1.397 (0.410-4.766) | 0.593 |
| *Quartile 3* | *65* | *1* | *0.094 (0.010-0.892)* | *0.039* |
| Quartile 4 | 65 | 8 | 1.179 (0.286-4.867) | 0.819 |
| Age |  |  |  |  |
| Below median | 126 | 11 | 1 (Ref.) |  |
| Above Median | 134 | 15 | 2.059 (0.872-4.864) | 0.100 |
| Coronary Heart Disease |  |  |  |  |
| Absent | 125 | 9 | 1 (Ref.) |  |
| Present | 135 | 17 | 1.584 (0.640-3.920) | 0.320 |
| Diabetes |  |  |  |  |
| Absent | 176 | 15 | 1 (Ref.) |  |
| Present | 84 | 11 | 1.812 (0.765-4.294) | 0.177 |
| Gender |  |  |  |  |
| Female | 68 | 9 | 1 (Ref.) |  |
| Male | 192 | 17 | 1.787 (0.557-5.739) | 0.329 |
| Hypertension |  |  |  |  |
| Absent | 57 | 5 | 1 (Ref.) |  |
| Present | 203 | 21 | 0.778 (0.255-2.376) | 0.659 |
| Smoking History |  |  |  |  |
| Absent | 34 | 2 | 1 (Ref.) |  |
| Present | 226 | 24 | 2.238 (0.488-10.262) | 0.300 |

HR = hazard ratio, CI = confidence interval, Ref. = reference. Relative visceral adipose volume = visceral-to-total abdominal adipose volume ratio. Quartiles are stratified by relative visceral adipose volume in ascending order. The significance level is 0.05. *Italicised* font indicates significance.
