# Supplementary material for: The Association of Visceral Adiposity with Cardiovascular Events in Patients with Peripheral Artery Disease
Source: PLoS One. 2013 Dec 27;8(12):e82350. doi: 10.1371/journal.pone.0082350 (PMC3873921; doi:10.1371/journal.pone.0082350)
Supplement: Table S2 — Independent determinants of non-fatal stroke in patients with PAD. (DOCX) [file pone.0082350.s008.docx]

**Table S2: Independent determinants of non-fatal stroke in patients with PAD.**

| **Prognostic Factor** | **Sample Size (n=260)** | **Stroke (n=10)** | **HR (95% C.I.)** | ***P* Value** |
| --- | --- | --- | --- | --- |
| Relative visceral adipose volume |  |  |  |  |
| Quartile 1 | 65 | 3 | 1 (Ref.) |  |
| Quartile 2 | 65 | 2 | 1.264 (0.156-10.263) | 0.827 |
| Quartile 3 | 65 | 3 | 2.217 (0.272-18.072) | 0.457 |
| Quartile 4 | 65 | 2 | 1.604 (0.129-19.916) | 0.713 |
| Age |  |  |  |  |
| Below median | 126 | 4 | 1 (Ref.) |  |
| Above Median | 134 | 6 | 1.801 (0.471-6.887) | 0.390 |
| Coronary Heart Disease |  |  |  |  |
| Absent | 125 | 5 | 1 (Ref.) |  |
| Present | 135 | 5 | 0.959 (0.240-3.837) | 0.953 |
| Diabetes |  |  |  |  |
| Absent | 176 | 7 | 1 (Ref.) |  |
| Present | 84 | 3 | 0.951 (0.223-4.055) | 0.946 |
| Gender |  |  |  |  |
| Female | 68 | 4 | 1 (Ref.) |  |
| Male | 192 | 6 | 3.214 (0.500-20.660) | 0.219 |
| Hypertension |  |  |  |  |
| Absent | 57 | 3 | 1 (Ref.) |  |
| Present | 203 | 7 | 0.587 (0.136-2.524) | 0.474 |
| Smoking History |  |  |  |  |
| Never | 34 | 0 |  |  |
| Current | 83 | 3 | 1 (Ref.)* | 0.981 |
| Ex-smoker | 133 | 7 | 1.434 (0.357-5.768)* | 0.612 |

HR = hazard ratio, CI = confidence interval, Ref. = reference. Relative visceral adipose volume = visceral-to-total abdominal adipose volume ratio. Quartiles are stratified by relative visceral adipose volume in ascending order. The significance level is 0.05. *Italicised* font indicates significance. *No patients with a history of never smoking suffered a stroke therefore this data was generated from a separate analysis excluding participants without a smoking history adjusted for the same risk factors. For the latter analysis smoking was defined as current or ex-smoking based on whether patients had smoked in the last month.
