# Supplementary material for: The Association of Visceral Adiposity with Cardiovascular Events in Patients with Peripheral Artery Disease
Source: PLoS One. 2013 Dec 27;8(12):e82350. doi: 10.1371/journal.pone.0082350 (PMC3873921; doi:10.1371/journal.pone.0082350)
Supplement: Table S3 — Independent determinants of cardiovascular events (myocardial infarction, stroke, death) in patients with PAD and diabetes mellitus. (DOCX) [file pone.0082350.s009.docx]

**Table S3: Independent determinants of cardiovascular events (myocardial infarction, stroke, death) in patients with PAD and diabetes mellitus.**

| **Prognostic Factor** | **Sample Size (n=84)** | **Cardiovascular Events (n=27)** | **HR (95% C.I.)** | ***P* Value** |
| --- | --- | --- | --- | --- |
| Relative visceral adipose volume |  |  |  |  |
| Quartile 1 | 21 | 5 | 1 (Ref.) |  |
| *Quartile 2* | *21* | *7* | *6.273 (1.295-30.390)* | *0.023* |
| *Quartile 3* | *21* | *8* | *4.865 (1.048-22.582)* | *0.043* |
| Quartile 4 | 21 | 7 | 3.256 (0.598-17.728) | 0.172 |
| Age |  |  |  |  |
| Below median | 47 | 13 | 1 (Ref.) |  |
| Above Median | 37 | 14 | 1.191 (0.529-2.681) | 0.673 |
| Coronary Heart Disease |  |  |  |  |
| Absent | 29 | 6 | 1 (Ref.) |  |
| Present | 55 | 21 | 1.485 (0.543-4.064) | 0.441 |
| Gender |  |  |  |  |
| Female | 18 | 7 | 1 (Ref.) |  |
| Male | 66 | 20 | 3.254 (0.850-12.459) | 0.085 |
| Hypertension |  |  |  |  |
| Absent | 11 | 5 | 1 (Ref.) |  |
| Present | 73 | 22 | 2.299 (0.571-9.247) | 0.241 |
| Smoking History |  |  |  |  |
| Absent | 14 | 3 | 1 (Ref.) |  |
| Present | 70 | 24 | 2.111 (0.571-7.809) | 0.263 |

HR = hazard ratio, CI = confidence interval, Ref. = reference. Relative visceral adipose volume = visceral-to-total abdominal adipose volume ratio. Quartiles are stratified by relative visceral adipose volume in ascending order. The significance level is 0.05. *Italicised* font indicates significance.
