# Supplementary material for: The Association of Visceral Adiposity with Cardiovascular Events in Patients with Peripheral Artery Disease
Source: PLoS One. 2013 Dec 27;8(12):e82350. doi: 10.1371/journal.pone.0082350 (PMC3873921; doi:10.1371/journal.pone.0082350)
Supplement: Table S4 — Independent determinants of cardiovascular events (myocardial infarction, stroke, death) in patients with PAD without diabetes mellitus. (DOCX) [file pone.0082350.s010.docx]

**Table S4: Independent determinants of cardiovascular events (myocardial infarction, stroke, death) in patients with PAD *without* diabetes mellitus.**

| **Prognostic Factor** | **Sample Size (n=176)** | **Cardiovascular Events (n=49)** | **HR (95% C.I.)** | ***P* Value** |
| --- | --- | --- | --- | --- |
| Relative visceral adipose volume |  |  |  |  |
| Quartile 1 | 44 | 17 | 1 (Ref.) |  |
| Quartile 2 | 44 | 12 | 1.076 (0.438-2.645) | 0.873 |
| Quartile 3 | 44 | 8 | 0.805 (0.283-2.289) | 0.684 |
| Quartile 4 | 44 | 12 | 0.827 (0.290-2.361) | 0.723 |
| Age |  |  |  |  |
| Below median | 79 | 11 | 1 (Ref.) |  |
| *Above Median* | *97* | *38* | *5.978 (2.649-13.490)* | *<0.001* |
| Coronary Heart Disease |  |  |  |  |
| Absent | 96 | 20 | 1 (Ref.) |  |
| Present | 80 | 29 | 1.780 (0.913-3.474) | 0.091 |
| Gender |  |  |  |  |
| Female | 50 | 19 | 1 (Ref.) |  |
| Male | 126 | 30 | 1.534 (0.656-3.586) | 0.323 |
| Hypertension |  |  |  |  |
| Absent | 46 | 9 | 1 (Ref.) |  |
| Present | 130 | 40 | 0.638 (0.279-1.461) | 0.288 |
| Smoking History |  |  |  |  |
| Absent | 20 | 4 | 1 (Ref.) |  |
| Present | 156 | 45 | 2.568 (0.841-7.841) | 0.098 |

HR = hazard ratio, CI = confidence interval, Ref. = reference. Relative visceral adipose volume = visceral-to-total abdominal adipose volume ratio. Quartiles are stratified by relative visceral adipose volume in ascending order. The significance level is 0.05. *Italicised* font indicates significance.
