# Supplementary material for: The Association of Visceral Adiposity with Cardiovascular Events in Patients with Peripheral Artery Disease
Source: PLoS One. 2013 Dec 27;8(12):e82350. doi: 10.1371/journal.pone.0082350 (PMC3873921; doi:10.1371/journal.pone.0082350)
Supplement: Table S5 — Independent determinants of cardiovascular events (myocardial infarction, stroke, death) in patients with AAA. (DOCX) [file pone.0082350.s011.docx]

**Table S5: Independent determinants of cardiovascular events (myocardial infarction, stroke, death) in patients with AAA.**

| **Prognostic Factor** | **Sample Size (n=136)** | **Cardiovascular Events (n=47)** | **HR (95% C.I.)** | ***P* Value** |
| --- | --- | --- | --- | --- |
| Relative visceral adipose volume |  |  |  |  |
| Quartile 1 | 34 | 17 | 1 (Ref.) |  |
| Quartile 2 | 34 | 10 | 0.514 (0.189-1.394) | 0.191 |
| Quartile 3 | 34 | 9 | 0.466 (0.165-1.318) | 0.150 |
| Quartile 4 | 34 | 11 | 0.439 (0.148-1.307) | 0.139 |
| Age |  |  |  |  |
| Below median | 46 | 10 | 1 (Ref.) |  |
| *Above Median* | *90* | *37* | *3.431 (1.532-7.684)* | *0.003* |
| Coronary Heart Disease |  |  |  |  |
| Absent | 61 | 19 | 1 (Ref.) |  |
| Present | 75 | 28 | 1.906 (0.953-3.811) | 0.068 |
| Diabetes |  |  |  |  |
| Absent | 108 | 35 | 1 (Ref.) |  |
| Present | 28 | 12 | 1.350 (0.597-3.053) | 0.471 |
| Gender |  |  |  |  |
| Female | 29 | 14 | 1 (Ref.) |  |
| Male | 107 | 33 | 1.165 (0.463-2.932) | 0.746 |
| Hypertension |  |  |  |  |
| Absent | 27 | 9 | 1 (Ref.) |  |
| Present | 109 | 38 | 0.694 (0.281-1.717) | 0.430 |
| Smoking History |  |  |  |  |
| Absent | 13 | 3 | 1 (Ref.) |  |
| Present | 123 | 44 | 2.195 (0.630-7.644) | 0.217 |

AAA was defined as an infra-renal aortic diameter ≥30mm. 26 patients were included who had lower limb athero-thrombosis as well as an AAA. HR = hazard ratio, CI = confidence interval, Ref. = reference. Relative visceral adipose volume = visceral-to-total abdominal adipose volume ratio. Quartiles are stratified by relative visceral adipose volume in ascending order. The significance level is 0.05. *Italicised* font indicates significance.
