# Supplementary material for: The Association of Visceral Adiposity with Cardiovascular Events in Patients with Peripheral Artery Disease
Source: PLoS One. 2013 Dec 27;8(12):e82350. doi: 10.1371/journal.pone.0082350 (PMC3873921; doi:10.1371/journal.pone.0082350)
Supplement: Table S6 — Independent determinants of cardiovascular events (myocardial infarction, stroke, death) in patients with PAD excluding AAA patients. (DOCX) [file pone.0082350.s012.docx]

**Table S6: Independent determinants of cardiovascular events (myocardial infarction, stroke, death) in patients with PAD *excluding AAA patients*.**

| **Prognostic Factor** | **Sample Size (n=124)** | **Cardiovascular Events (n=29)** | **HR (95% C.I.)** | ***P* Value** |
| --- | --- | --- | --- | --- |
| Relative visceral adipose volume |  |  |  |  |
| Quartile 1 | 31 | 8 | 1 (Ref.) |  |
| Quartile 2 | 31 | 6 | 0.937 (0.291-3.022) | 0.914 |
| Quartile 3 | 31 | 6 | 2.408 (0.613-9.462) | 0.208 |
| Quartile 4 | 31 | 9 | 2.682 (0.591-12.167) | 0.201 |
| Age |  |  |  |  |
| Below median | 80 | 14 | 1 (Ref.) |  |
| Above Median | 44 | 15 | 1.934 (0.864-4.328) | 0.109 |
| Coronary Heart Disease |  |  |  |  |
| Absent | 64 | 7 | 1 (Ref.) |  |
| Present | 60 | 22 | 2.272 (0.874-5.904) | 0.092 |
| Diabetes |  |  |  |  |
| Absent | 68 | 14 | 1 (Ref.) |  |
| Present | 56 | 15 | 1.044 (0.471-2.312) | 0.916 |
| Gender |  |  |  |  |
| Female | 39 | 17 | 1 (Ref.) |  |
| Male | 85 | 12 | 2.999 (0.957-9.396) | 0.059 |
| Hypertension |  |  |  |  |
| Absent | 30 | 5 | 1 (Ref.) |  |
| Present | 94 | 24 | 0.798 (0.274-2.321) | 0.678 |
| Smoking History |  |  |  |  |
| Absent | 21 | 4 | 1 (Ref.) |  |
| Present | 103 | 25 | 1.275 (0.403-4.032) | 0.680 |

HR = hazard ratio, CI = confidence interval, Ref. = reference. Relative visceral adipose volume = visceral-to-total abdominal adipose volume ratio. Quartiles are stratified by relative visceral adipose volume in ascending order. The significance level is 0.05.
